# The Smoke We Breathe

**Published:** 1924-12

**Authors:** 


### THE SMOKE WE BREATHE.

The English are a remarkable people. The more they talk about a subject, the less they do. They are content to weep for years over a difficulty or a danger, and so enamoured do they become of their tears that they are reluctant to dry them by the obvious process of removing the trouble. Thus it is with the eternal subject of smoke abatement. We have a Smoke Abatement League and thousands of doctors and scientists who support it and make speeches pointing out the loss in health, efficiency and money caused by the thick veil which, on most days of the year, we allow to come between us and the beneficent face and heating rays of the sun. Yet year succeeds year and, notwithstanding spasmodic legislation, very little is done. Steady attrition is, nevertheless, a powerful agent. The drop of water falling upon the stone wears it away at last, and we must hope that, in the end, all the speeches and articles will awaken the country to the fact that it is injuring its health, degenerating its physique, and wasting its money by continuing to permit the atmosphere to be polluted.

This much-desired day should be hastened, at all events in the North of England, by the exhibition of appliances for diminishing smoke which has just been held at Manchester, and by the illuminating statistics there displayed by the Smoke Abatement League to which we owe this useful demonstration. To those who are not closely familiar with the subject, the League's figures will be startling. They show that the soot-fall per square mile per annum in Newcastle-upon-Tyne is 885 tons, in Rochdale 794, in Birmingham 440, in London 314. One of the objects of the exhibition was to fasten as much guilt as possible upon the open domestic fire; the other was a protest against the ordinary wasteful method of burning coal in factory furnaces.

The enormously greater proportion of soot in manufacturing towns clearly suggests that it is in the factory that reform should begin. There are now many effective appliances for the prevention of factory smoke, and certain legislation already exists under which owners of great chimney-stacks are precluded from emitting black smoke. But we need only to approach a large industrial town to realise that the law is, in great measure, a dead letter. There, at all events, the domestic fire counts for little. But that elsewhere it does count, and count considerably, is not to be denied. Reform lies, however, not in the abolition of the open fire but in the use of a smokeless fuel or the gradual adoption of some such methods of smoke consumption as have been found more or less effective in factories. A campaign against the Englishman's cherished open fire is an almost hopeless enterprise. Nor does there seem to be any conclusive reason for depriving him of it. Moreover, he is always able to retort upon the reformers that residential towns enjoy a comparatively clean atmosphere, and that it is only the manufacturing centres in which the air is badly polluted. But in time we shall learn that it need not be polluted anywhere, and that, if we are entitled to the traditional hearty blaze, we are also entitled to bask in the curative and preventive rays of the sun.

